# Microbial dysbiosis and wound healing in diabetic foot ulcers: a mini review with a note on the role of artificial intelligence

**DOI:** 10.3389/fcimb.2026.1891884

**Published:** 2026-07-15

**Authors:** Xinxin Yu, Qi Liu, Yanhong Huang, Qian Wang, Ye Wang, Gang Zhao

**Affiliations:** 1 First Clinical Medical College, Heilongjiang University of Chinese Medicine, Harbin, China; 2 Department of Peripheral Vascular II, First Affiliated Hospital of Heilongjiang University of Chinese Medicine, Harbin, China

**Keywords:** artificial intelligence, biofilm, diabetic foot ulcer, gut-skin axis, microbiome dysbiosis, phage therapy

## Abstract

Diabetic foot ulcers (DFUs) are a serious diabetes-related complication characterized by high rates of amputation and mortality. Emerging evidence suggests that DFUs are not simply the result of infection, but also involve microbiome dysbiosis, which impairs healing. Systemically, disturbances to the gut microbiota via the gut-skin axis promote systemic inflammation and metabolic dysfunction. Locally, skin microbial diversity is significantly reduced, allowing opportunistic pathogens such as *Staphylococcus aureus* and *Pseudomonas aeruginosa* to form resilient biofilms. These biofilms resist antibiotics and host immunity, while microbial virulence factors exacerbate tissue damage and disrupt the healing cascade. This synergy between host pathology and dysbiosis perpetuates chronic ulceration. Novel therapeutic strategies therefore aim to modulate this aberrant ecology by shifting from broad-spectrum eradication to targeted restoration. Promising approaches include probiotics, phage therapy, traditional Chinese medicine, and faecal microbiota transplantation, which seek to recalibrate the microbiome and promote healing. However, translation into clinical practice requires more robust evidence from large-scale trials. Future perspectives point towards personalized microbial medicine, integrating multi-omics data and artificial intelligence to match interventions with specific microbial ecotypes, which may reduce the global burden of DFUs.

## 1 Introduction

Diabetic foot ulcers (DFUs) are a severe chronic complication of diabetes and are characterized by a combination of vasculopathy, neuropathy, and infection (Bus et al., 2024). This condition imposes a substantial global health burden, with an estimated lifetime risk of 19–34%, a prevalence of around 6.3% among adults with diabetes and a lifetime incidence of lower-extremity amputation of 20% (McDermott et al., 2023). DFUs are the leading cause of non-traumatic lower-extremity amputations, are associated with high rates of recurrence and hospitalization, and have a 5-year mortality rate of 50-70%. However, this rate increases to over 70% for those undergoing major amputation (Pavithra and Kaliyaperumal, 2026). Furthermore, the annual mortality rate among individuals with DFUs is 231 deaths per 1,000 person-years, which is significantly higher than the rate of 182 deaths per 1,000 person-years observed in diabetic patients without foot ulcers. Thus, the management of DFUs poses considerable clinical and socioeconomic challenges, highlighting the urgent need for advanced therapeutic strategies (Armstrong et al., 2023).

For decades, the microbial dimension of DFUs has predominantly been viewed through the lens of infection, focusing on the identification and eradication of specific pathogens (Kalan et al., 2019). However, this perspective fails to fully explain the chronicity and therapeutic recalcitrance that are characteristic of many ulcers, in which standard antimicrobial therapies often yield suboptimal results. Contemporary understanding reframes these wounds not merely as site of infection, but as dysbiotic wound microenvironments. Evidence suggests that DFUs are often associated with local skin microbiome dysbiosis, characterized by a reduction in beneficial microbial diversity and an increase in opportunistic pathogens (Lou et al., 2025). This dysbiosis is an active driver of pathology that disrupts the normal wound healing cascade. Furthermore, emerging research highlights the pivotal role of gut microbiome dysbiosis in this process. Through systemic pathways such as the gut-skin axis, intestinal microbial imbalance can exacerbate inflammation and impair immune regulation, thereby contributing to the non-healing phenotype (Jimenez-Sanchez et al., 2025). Thus, the pathological microbial landscape of DFUs is increasingly understood as an interplay between disrupted skin and gut microbiomes, which together sustain chronicity and complicate treatment. This integrated perspective highlights the need for therapeutic strategies that address both local and systemic dysbiosis in order to effectively promote wound healing.

Multi-omics technologies have been widely applied to profile DFU-associated microbiomes, yet conventional analytical methods struggle to interpret high-dimensional datasets and guide personalized treatment (Lou et al., 2025). Artificial intelligence (AI) has become a powerful tool for mining complex microbiome data, predicting pathogen traits and treatment responses (Omo-Okhuasuyi et al., 2024; Lu et al., 2025), while relevant comprehensive reviews focusing on AI and DFU microbiome remain scarce. This mini-review summarizes current understanding of how microbiome dysbiosis disrupts healing processes in DFUs, evaluates the therapeutic potential of targeted microbial modulation, and further discusses the application prospects of AI in this field.

This mini-review synthesizes current evidence on microbiome dysbiosis and wound healing in diabetic foot ulcers from peer-reviewed original studies, systematic reviews/meta-analyses, and relevant clinical reports. Literature was identified through searches of PubMed, Web of Science, and Scopus, prioritizing publications from approximately the past decade and focusing on English-language articles. Search terms centered on diabetic foot ulcer/diabetic foot, microbiome/microbiota/dysbiosis/biofilm, and related themes (gut-skin axis, microbial modulation, probiotic/phage/FMT, AI-multi-omics). Citations were further expanded by backward-forward tracing of key reviews and guideline references. No formal systematic review protocol or PRISMA flow was applied; instead, studies were selected based on topical relevance, mechanistic/clinical importance, and the strength of available evidence, with an emphasis on distinguishing preclinical findings, small-scale clinical data, and more robustly replicated conclusions.

The systemic implications of dysbiosis mediated by the gut-skin axis are first discussed, as they may predispose affected individuals to impaired cutaneous wound responses. Local pathological mechanisms driving DFU chronicity are subsequently analyzed, spanning from biofilm-associated therapeutic resistance to the dysregulation of host immune cell function. Finally, emerging therapeutic strategies and AI-enabled precision management for precise recalibration of the wound microbiome are critically appraised. These approaches advance beyond conventional broad-spectrum antimicrobials to interventions that restore a pro-healing microbial ecosystem.

## 2 The pathogenesis of diabetic foot ulcers

The pathogenesis of DFUs is complex, involving interactions among multiple pathophysiological processes. In recent years, researchers have explored the development and progression of DFUs from various perspectives, including multi-omics, immunology, metabolic regulation, and molecular signaling pathways. The following systematic review focuses on five core aspects.

### 2.1 Vascular pathology and microenvironmental disturbances

Patients with DFUs often present with significant vascular pathology, characterized by microvascular dysfunction, reduced angiogenic capacity, and tissue ischemia. Hyperglycemia is the primary driving factor, disrupting the vascular microenvironment through multiple pathways. Hyperglycemia drives the *de novo* synthesis of diacylglycerol, which activates the protein kinase C (PKC) pathway. This leads to endothelial dysfunction, increased vascular permeability and reduced nitric oxide production (Liu et al., 2024). Simultaneously, hyperglycemia promotes the accumulation of advanced glycation end products (AGEs), which bind to their receptor RAGE. This triggers persistent inflammation and reactive oxygen species (ROS) generation. It also activates downstream signaling pathways, such as NF-κB and MAPK. This directly damages endothelial cells and extracellular matrix (ECM) proteins (Qaed et al., 2025). Furthermore, the expression of hypoxia-inducible factor 1α (HIF-1α), a key transcription factor, is reduced in diabetic wounds. This leads to decreased expression of its target genes such as vascular endothelial growth factor (VEGF) and fibroblast growth factor 2. This impairs angiogenesis and delays wound healing (Brocato et al., 2014).

### 2.2 Neuropathy and peripheral nerve injury

Diabetic peripheral neuropathy is a major cause of DFUs. Hyperglycemia activates the polyol pathway, leading to the accumulation of sorbitol and fructose in neuronal cells. This causes osmotic stress and reduces myo−inositol synthesis, thereby decreasing nerve conduction velocity (Garg and Gupta, 2022). Loss of protective sensation means that patients cannot perceive foot trauma, while motor neuropathy causes foot muscle atrophy and deformities. This results in abnormal plantar pressure distribution, increasing the risk of ulceration. Autonomic neuropathy leads to anhidrosis of the lower limbs and dry, cracked skin, which provides a portal for bacterial invasion (Dawi et al., 2025). Together, these neuropathic changes increase the risk of trauma and delay wound healing.

### 2.3 Metabolic disorders and mitochondrial dysfunction

Metabolic disorders drive DFUs through multiple interconnected mechanisms. Firstly, high glucose levels can lead to mitochondrial dysfunction, which is characterized by an increase in ROS, reduced ATP synthesis, disrupted calcium homeostasis, abnormal autophagy, and ferroptosis. This can impair repair cells and delay tissue regeneration (Pan et al., 2025). Secondly, the accumulation of branched-chain amino acids (BCAAs) activates the mTORC1/NF-κB pathway, resulting in insulin resistance, M1 macrophage polarization, and impaired angiogenesis. This forms a metabolic−immune−vascular vicious cycle (Tang et al., 2026). Thirdly, increased lactylation, alongside decreased expression of CHD4, EEF1A1, and EEF1G, disrupts the immune microenvironment (Hu et al., 2026). Concurrently, phenylpyruvate accumulation in DFUs activates the NLRP3 inflammasome, which drives M1 macrophage polarization and pro−inflammatory cytokine release. This further reinforces a metabolic−inflammatory vicious cycle that synergizes with the BCAAs−mediated pathway (Lv et al., 2023).

### 2.4 Peripheral artery disease (PAD) and ischemic injury

PAD is an important comorbidity of DFUs. The ischemic state resulting from both PAD and microvascular dysfunction is closely associated with poor DFUs healing. The vascular pathology in diabetic patients is characterized by macro− and microvascular atherosclerosis, which is promoted by hyperglycemia, dyslipidemia, insulin resistance, and inflammation (Zakir et al., 2023). Ischemia further impairs microcirculation, reducing the supply of oxygen and nutrients required for wound healing. It is also linked to complications such as infection and osteomyelitis (Pham et al., 2025). When neuropathic foot deformity and abnormal pressure are superimposed on the lack of repair resources caused by ischemia, even minor trauma can evolve into a chronic ulcer (Cao et al., 2022).

### 2.5 Inflammatory dysregulation and immune microenvironment abnormalities

**Figure 1:**
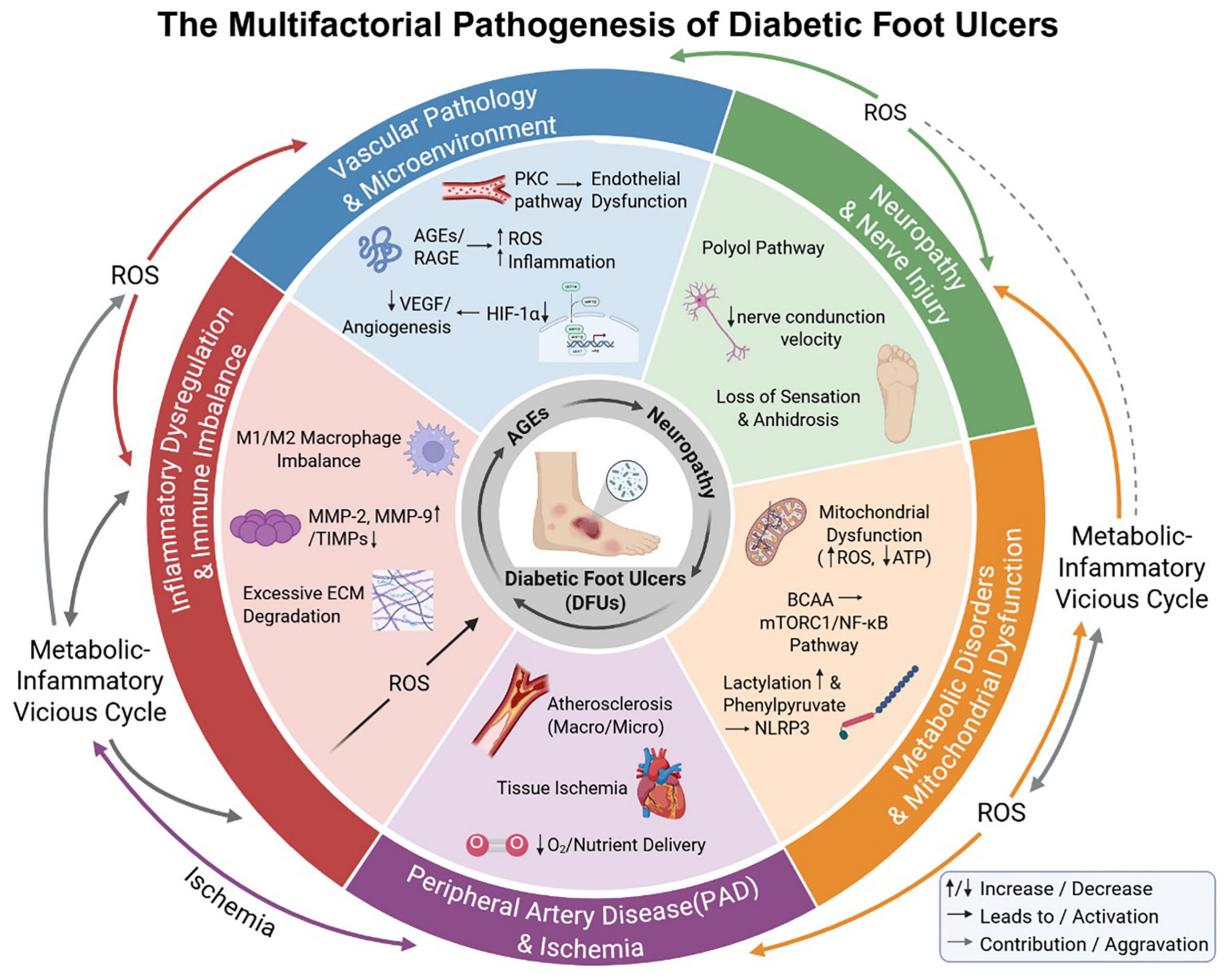
Pathogenesis of diabetic foot ulcers.

The chronic non−healing nature of DFUs is fundamentally attributed to a persistent inflammatory state. Key mechanisms include the following. First, impaired macrophage phenotype switching: in diabetic wounds, persistent oxidative stress and AGEs accumulation under hyperglycemic conditions maintain the presence of pro−inflammatory M1 macrophages, while the transition to reparative M2 macrophages is blocked (Feng et al., 2022). The insufficient secretion of growth factors such as IL−10, TGF−β, and VEGF by M2 macrophages, coupled with the sustained release of TNF−α, IL−1β, and ROS by M1 macrophages, collectively prevents the wound from progressing from the inflammatory phase to the proliferation and remodeling phases (Wolf et al., 2021; Wang et al., 2026). Second, an imbalance between matrix metalloproteinases (MMPs) and their tissue inhibitors (TIMPs): in diabetic wounds, the activities of MMP−2 and MMP−9 are significantly elevated, while TIMPs expression is reduced. This leads to excessive degradation of the ECM (Chen et al., 2023). This abnormal proteolytic environment destroys the ECM framework necessary for wound healing, thereby delaying wound closure. **Figure 1**.

## 3 Gut-skin dysbiosis in diabetic foot ulcers

The impaired healing of DFUs is increasingly understood within the framework of systemic host-microbiota interactions, notably through the conceptual model of the “gut-skin axis” (Patel et al., 2022; Huang et al., 2025). This theory describes the bidirectional, systemic communication between the gut microbiota and distant skin, which is mediated by microbial metabolites, immune signaling, and endocrine pathways (Patel et al., 2022).

### 3.1 Gut microbiota dysbiosis in diabetic foot ulcers

Gut microbiota dysbiosis can initiate a detrimental cascade of “gut microbiota–systemic inflammation–impaired skin repair.” Studies confirm that specific gut microbiota, such as an increased abundance of the *Streptococcus genus*, is positively correlated with the occurrence of DFUs in patients (Wang et al., 2022). This suggests that disturbances in the gut microbial ecosystem and its metabolic output are key systemic factors that drive the development and progression of chronic diabetic wounds, providing a core pathophysiological basis for the existence of the “gut-skin axis” (Huang et al., 2025).

Animal studies and preliminary human data suggest that gut microbiota dysbiosis may mediate the chronicity of DFUs through several interconnected signaling pathways. However, direct causal evidence in humans remains limited. Firstly, disruption of the microbial metabolome is a key mechanism. On the one hand, gut dysbiosis leads to the systemic accumulation of metabolites such as BCAAs, partly due to the downregulation of the key BCAAs catabolic enzyme, BCKDH, in peripheral tissues, such as skeletal muscle (Tang et al., 2026). Preclinical evidence indicates that BCAAs can persistently activate the mTORC1/NF-κB signaling axis, thereby triggering insulin resistance, mitochondrial dysfunction, oxidative stress and the polarization of macrophages towards a pro-inflammatory M1 phenotype. Together, these events impair angiogenesis mediated by the HIF-1α/VEGF pathway, forming a self-perpetuating “metabolic-immune-vascular” vicious cycle that delays wound healing (Tang et al., 2026). On the other hand, the deficiency of key beneficial metabolites is equally important. In diabetic patients, for example, the production of short-chain fatty acids (SCFAs) by the gut microbiota is typically reduced. SCFAs possess important anti-inflammatory and immune homeostasis-maintaining functions. Their deficient production weakens these regulatory effects, thereby directly linking abnormal gut metabolic output to impaired skin repair function (Huang et al., 2025). Secondly, the existence of a “gut microbiota-inflammation-skin” axis has been experimentally validated in rodent models. Research shows that the bioactive component of traditional Chinese medicine (TCM), quercetin, can accelerate diabetic wound healing by modulating the gut microbiota, reducing systemic inflammatory factors, including TNF-α and IL-1β, and enhancing local wound angiogenesis. Thirdly, specific microbiota compositions are associated with disease severity and predicted pathogenic functions. Compared to healthy controls or patients with mild DFUs, the microbiota of patients with severe DFUs is characterized by an increased proportion of bacterial taxa such as *Bacteroidetes*, *Prevotella*, *Porphyromonas*, and *Dialister (*Park et al., 2019). Functional predictive analysis indicates that the gene functions of these microbiotas associated with severe wounds are enriched in pathways such as lipopolysaccharide biosynthesis, pro-inflammatory cellular antigen production, and protein digestion metabolism. This suggests that they may worsen DFUs prognosis by exacerbating inflammation and directly causing tissue damage (Park et al., 2019).

The systemic immune disorder caused by gut dysbiosis also creates conditions that lead to infection and chronicity of DFUs. Imbalance in the gut microbiota disrupts systemic immune homeostasis, thereby increasing skin susceptibility to infection. In diabetic patients with pre-existing immune deficiencies and metabolic abnormalities, this state can cause skin-dwelling commensal bacteria (such as *Staphylococcus aureus*) to become invasive pathogens (Dörr et al., 2021; Dörr et al., 2023; Zhang et al., 2025). These pathogens readily form biofilms, resisting host immunity and antibiotics and leading to persistent, non-healing infections (Suryaletha et al., 2018). Research has also found that *Helcococcus kunzii*, another skin commensal that is often co-isolated with *S. aureus* from DFUs, may modulate staphylococcal virulence through potential quorum-sensing mechanisms, thereby exacerbating local infection (Durand et al., 2022). Meanwhile, persistent inflammatory signals originating from the gut exacerbate the pro-inflammatory microenvironment in foot ulcers. This is characterized by elevated levels of cytokines such as IL-6, TNF-α, and CRP, as well as neutrophil dysfunction (Sharma et al., 2022; Deng et al., 2024; Wang et al., 2025), which further hinders the healing process.

### 3.2 Skin microbiota dysbiosis in diabetic foot ulcers

The skin microbiome is a community of microorganisms that reside on the surface of the skin. They maintain a symbiotic relationship with the host and play an indispensable role in preserving the physical barrier, modulating local and systemic immunity, and competitively inhibiting the colonization of foreign pathogens (Lou et al., 2025). In a healthy state, the skin microbiome is highly diverse and relatively stable. It is predominantly composed of commensal bacteria such as *Staphylococcus epidermidis*, *Corynebacterium, Propionibacterium, Micrococcus, and Malassezia* (Prajapati et al., 2025). These resident microbes engage in mutualism with the host through various mechanisms. For instance, they secrete antimicrobial peptides that inhibit the growth of pathogens like *S. aureus*, thereby maintaining skin homeostasis (MacGibeny et al., 2025). In DFUs, however, this delicate microecological balance is profoundly disrupted, with the most notable hallmark being a significant loss of overall microbial diversity (Lou et al., 2025). This reduction in diversity is often associated with poorer clinical prognoses. The vacated ecological niche is occupied by a limited consortium of opportunistic pathogens, leading to a state of typical dysbiosis. DFUs infections are usually caused by multiple bacteria, and the specific composition of the infection is influenced by various factors, including geographical location, ulcer duration, history of antibiotic use, and ulcer severity (Prajapati et al., 2025). From a global epidemiological perspective, *S. aureus* is the most frequently isolated pathogen, followed by *Pseudomonas aeruginosa*, *Escherichia coli*, *Enterococcus* spp., and *Proteus* spp (Macdonald et al., 2021; Zhou et al., 2024). Multidrug-resistant organisms such as methicillin-resistant *S. aureus* (MRSA) and Gram-negative bacteria that produce extended-spectrum beta-lactamase are prevalent in DFUs. They are significantly associated with delayed wound healing, an increased risk of recurrence, and higher rates of lower extremity amputation (Woldeteklie et al., 2022; Zhou et al., 2024).

Arguably, biofilm formation is the most critical pathological mechanism driving the transition of DFUs from acute injuries to chronic, non-healing wounds. Biofilms are present in the majority of DFUs, with studies implicating them in around 80% of chronic cases (Zafer et al., 2024). A biofilm is an extracellular polymeric (EPS) matrix composed of polysaccharides, proteins, extracellular DNA, and lipids, which provides a powerful shelter for microorganisms (Zafer et al., 2024). Biofilms create a stubborn barrier to healing through multiple mechanisms. Firstly, the dense EPS matrix acts as an efficient physical and chemical barrier, severely impeding the penetration of antimicrobial agents and host immune cells such as neutrophils and macrophages. This renders conventional topical and systemic antibiotic therapies largely ineffective (Shrestha et al., 2022; Sahoo and Meshram, 2024). Secondly, bacteria within the biofilm often enter a state of reduced metabolic activity and slowed growth, which further increases their tolerance to antibiotics that typically target actively dividing cells (Sharma et al., 2019; Sahoo and Meshram, 2024).

In excess of the shelter provided by biofilms, dominant pathogens colonizing ulcers can directly damage tissue by expressing specific virulence factors, further subverting the host immune response. For instance, *S. aureus* produces pore-forming toxins such as alpha-toxin, which lyse host cell membranes and destroy keratinocytes and immune cells (Wang et al., 2025). *P. aeruginosa* secretes elastase, exotoxin A, as well as other factors that degrade the ECM growth factors that are crucial for wound repair. This exacerbates local inflammatory responses (Wang et al., 2025). Against the backdrop of pre-existing immune dysfunction in diabetes, the dysbiotic microbiome exploits and amplifies this defect. Pathogens and their products can impair neutrophil chemotaxis, phagocytosis, and bactericidal function, as well as promoting premature apoptosis of immune cells (Wang et al., 2023; Gan et al., 2024). Concurrently, microbial proteases can degrade key healing signaling molecules and disrupt the balance between matrix metalloproteinases and their inhibitors, leading to excessive ECM degradation (Mihai et al., 2024). Together, these processes collectively contribute to the failure of re-epithelialization and wound healing.

## 4 Novel therapeutic strategies targeting the microbiome and host environment

### 4.1 Probiotic and postbiotic therapies

Probiotics have the potential to effectively manage DFUs by competitively inhibiting pathogens, secreting antimicrobial metabolites such as organic acids and bacteriocins, and modulating the immune system via the “gut-skin axis.” (Srivastava et al., 2022; Çelo et al., 2026; Karmińska et al., 2026). Preliminary clinical evidence suggests that taking a specific probiotic mixture containing *Lactobacillus fermentum*, *Lactobacillus casei*, *Lactobacillus acidophilus*, and *Bifidobacterium bifidum* orally for 12 weeks significantly reduces ulcer dimensions and improves glycemic and cholesterol levels (Hussain et al., 2025). Regarding topical application, *Lactobacillus plantarum* has shown promising results: one study of chronically infected leg ulcers found that 43% of diabetic patients and 50% of non-diabetic patients achieved complete wound healing within 30 days (Awasthi et al., 2022). Another study indicated that topical application combined with surgical debridement serves as an effective adjuvant therapy (Argañaraz Aybar et al., 2022). Postbiotics, including heat-killed *Lactobacillus plantarum* GMNL-6 and *Lactobacillus paracasei* GNNL 653, have also been found to prevent excessive fibrosis and promote healing in animal models (Tsai et al., 2021). However, current evidence is primarily derived from small-scale studies, and a significant deficit of large-scale randomized controlled trials remains.

Despite these encouraging findings, several limitations temper the translational potential of probiotic therapy. Most studies are small, uncontrolled, or lack standardized formulations. The viability and stability of live probiotics in wound environments, potential risks of infection in immunocompromised patients, and the absence of regulatory guidelines for topical probiotic products represent major hurdles. Rigorous dose-finding and safety assessments are urgently needed.

### 4.2 Phage therapy

Phage therapy offers a promising alternative for managing multidrug−resistant bacteria, such as MRSA and *P. aeruginosa*, and their resilient biofilms in DFUs. A systematic review of 21 studies confirmed its efficacy against key pathogens including *S. aureus*, *P. aeruginosa*, *Enterococcus faecalis*, and *Klebsiella pneumoniae (*Esfandiari et al., 2025a). Phage cocktails have been shown to be more effective than single phages at preventing bacterial regrowth and overcoming resistance (Esfandiari et al., 2025b). A synergistic effect is observed when phages are combined with antibiotics such as ciprofloxacin, which enhances biofilm penetration and bacterial clearance (Liu et al., 2023). A case series involving infected toe ulcers caused by *S. aureus* demonstrated improvement in all cases following the weekly topical application of the phage Sb-1, with ulcers healing on average within 7 weeks (Plumet et al., 2025). In another series, nine out of ten patients with severe infections improved after adding anti-*S. aureus* phage therapy, with six achieving complete infection resolution. Notably, in cases of polymicrobial infection, clinical remission was achieved in four out of five patients, even when therapy targeted only the primary pathogen (Young et al., 2023). Phage therapy is generally well tolerated, but current evidence is primarily derived from case studies, representing a low level of evidence.

Nevertheless, the widespread clinical adoption of phage therapy for DFUs is constrained by several barriers. These include the lack of standardized phage preparation protocols, regulatory uncertainty, the narrow host range requiring personalized formulation, and the absence of large-scale randomized controlled trials to confirm efficacy and safety. Thus, while promising, phage therapy remains an experimental adjunct rather than a proven clinical strategy.

### 4.3 Faecal microbiota transplantation (FMT) in DFUs

Although high-quality clinical evidence for the direct application of FMT in DFUs remains scarce, mechanistic studies provide a solid experimental foundation for the “gut-skin axis” theory. A seminal animal study demonstrated that FMT promotes wound healing in type 2 diabetic mice via the IL-17A-mTOR-HIF1α signaling axis (Peng et al., 2025). Specifically, FMT corrected gut dysbiosis in diabetic mice by increasing the abundance of beneficial bacteria and reducing harmful bacteria. This microbial reconstruction promoted IL-17A expression, which subsequently activated AKT and mTOR signaling, thereby upregulating HIF-1α and its downstream glycolytic rate-limiting enzyme HK2 to provide energy for wound repair. Experimental data showed that the wound closure rate, re-epithelialization and collagen deposition were significantly higher in the FMT group than in the diabetic model group. The pro-healing effect of FMT was markedly attenuated when the IL-17A inhibitor Secukinumab was used to block this pathway, confirming the central role of this signaling axis (Peng et al., 2025). Further research indicates that FMT from quercetin-treated rats can also promote wound repair by modulating the microbiota and reducing systemic inflammation. These findings substantiate the potential of FMT to break the vicious cycle of systemic inflammation, local immune dysregulation, and impaired healing through ecological regulation (Huang et al., 2026). However, large-scale randomized controlled trials are needed to verify its efficacy and long-term safety before it can be used clinically.

It must be emphasized that all current FMT evidence for DFUs derives from rodent models. No human trial has yet evaluated FMT specifically for DFU healing. Critical unknowns include donor selection criteria, optimal administration route (oral capsule vs. enema), dosing frequency, and the potential for adverse transmission of pathogenic microorganisms. Until these gaps are addressed, FMT remains a purely investigational approach for DFU management.

### 4.4 Traditional Chinese medicine modulating gut and skin microbiota

Certain TCM formulations and herbal agents exert pro-healing effects by modulating the “gut-skin axis”. Specific TCM formulas and single herbs, such as Astragalus membranaceus, Paeonia lactiflora, Jin-Huang-San and Si-Miao-Yong-An-Tang, have been shown to increase SCFAs production, reduce serum pro−inflammatory cytokines (IL−1β and TNF−α) and enhance intestinal barrier proteins (ZO−1 and claudin−1). This alleviates systemic inflammation and promotes DFUs healing (Zhou et al., 2025). Research indicates that quercetin, a key active ingredient found in *Evodia rutaecarpa*, improves diabetic wound healing by modulating the structure of gut microbiota. In diabetic rat models, quercetin accelerated wound closure, improved glucolipid metabolism and reduced serum levels of TNF-α and IL-1β. This is achieved by directly binding to and stabilizing the HIF1α protein, thereby upregulating the HIF1α/VEGF pathway to enhance angiogenesis (Huang et al., 2026). Adjunctive therapies such as acupuncture (for example, surrounding needling, moxibustion) may also improve DFU outcomes by activating the vagus nerve−mediated anti−inflammatory pathway and enhancing lower limb microcirculation, partly via increased SCFAs generation (Zhou et al., 2025; Tang et al., 2026). These findings highlight the potential of TCM as a holistic, microbiome−modulating strategy in DFUs management, though more rigorous clinical trials are required.

**Figure 2:**
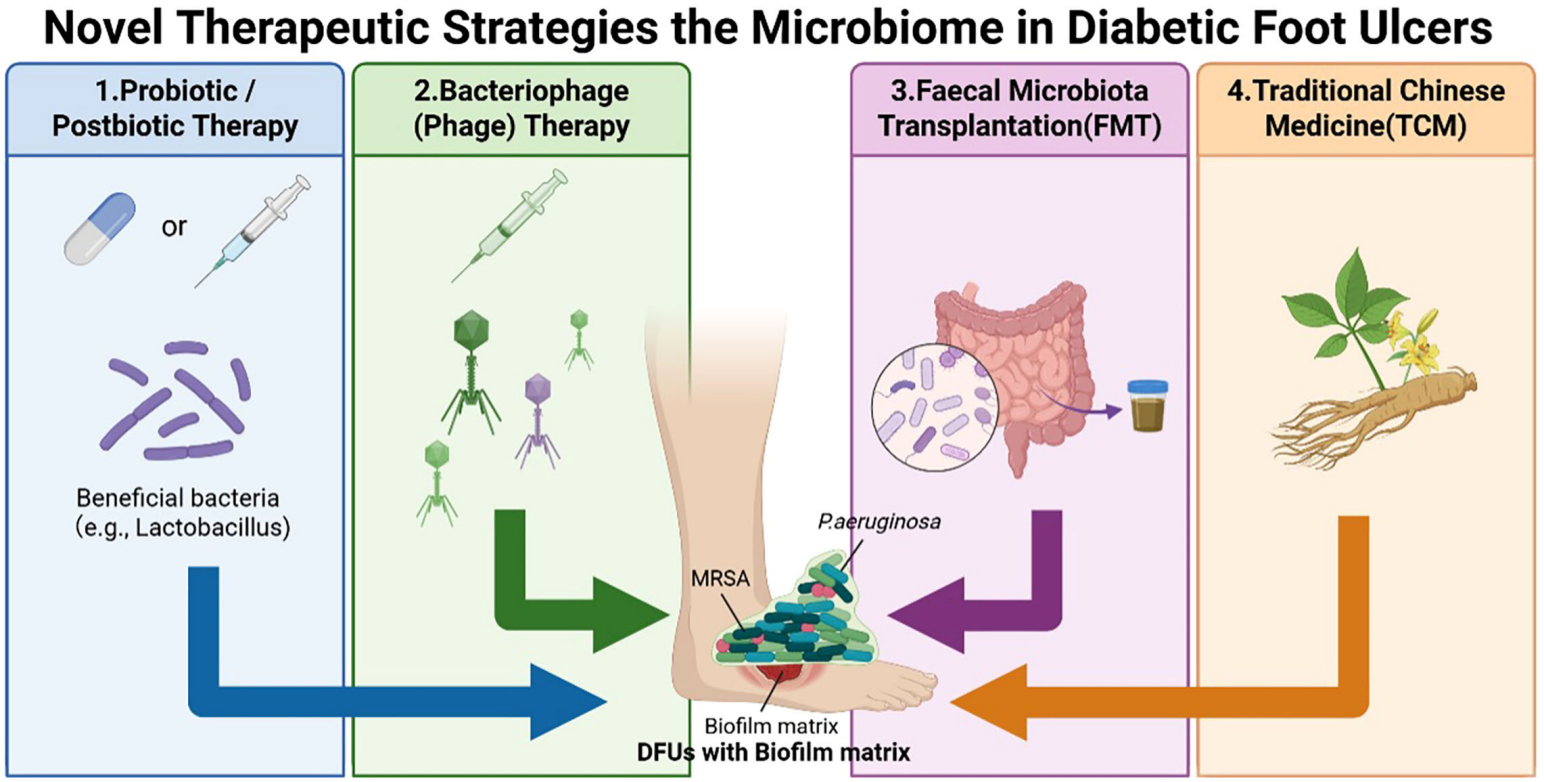
Therapies of diabetic foot ulcers.

Despite historical use and promising preclinical data, TCM faces significant challenges for evidence-based integration into DFU care. The complexity of multi-herb formulas makes standardization difficult; batch-to-batch variability, unclear active constituents, and potential herb-drug interactions are unresolved issues. Moreover, most clinical studies are small, lack blinding, and use heterogeneous outcome measures. High-quality randomized controlled trials with standardized extracts and rigorous endpoints are essential before TCM can be recommended as a mainstream therapy. **Figure 2**.

## 5 Artificial intelligence: advancing DFU microbiome research and precision management

The complex DFU microbiome and high-dimensional multi-omics data pose great challenges to traditional analytical methods (Guan et al., 2024). AI, especially machine learning and deep learning, has become a powerful tool to tackle these issues and drive precision treatment. AI algorithms can efficiently mine microbial signatures from metagenomic and metabolomic data, identify microbial biomarkers linked to ulcer severity, healing prognosis and amputation risk, and stratify patients into distinct microbial ecotypes accurately in controlled research settings (Omo-Okhuasuyi et al., 2024; Soldevila-Boixader et al., 2025). In biofilm and antimicrobial resistance research, AI models rapidly predict biofilm formation ability of dominant pathogens and antibiotic resistance profiles, greatly shortening detection time compared with conventional tests and guiding rational antibiotic use (T N et al., 2025; Carrillo-Larco et al., 2026).

Furthermore, AI optimizes microbiome-targeted therapies for DFUs (Hsiu-Jung et al., 2026). By analyzing individual gut and wound microbial profiles, machine learning models can design personalized probiotic formulas and phage cocktails, moving beyond the universal treatment mode and improving therapeutic efficacy (Hussain et al., 2025). AI also predicts patient responses to FMT, TCM and other interventions, helping clinicians select optimal regimens and avoid ineffective treatments (Lu et al., 2025). In clinical practice, AI-powered image analysis and decision support systems realize real-time wound monitoring and auxiliary diagnosis, bridging the gap between basic microbiome research and clinical application (Vijay et al., 2024; Nie et al., 2026).

Despite its promising prospects, the popularization of AI in DFU management still faces limitations. First, most existing AI models are trained on small, single-center datasets with limited external validation, raising concerns about generalizability across different populations and clinical settings. Second, the “black-box” nature of many deep learning algorithms hampers clinical trust and regulatory approval; explainable AI frameworks are urgently needed. Third, the integration of multi-omics data remains technically challenging due to heterogeneity in data formats, missing values, and batch effects. Fourth, real-world implementation is hindered by the lack of standardized data-sharing infrastructure, interoperability with electronic health records, and prospective clinical trials demonstrating that AI-guided decisions improve patient outcomes compared to standard care. Without addressing these validation and deployment challenges, AI tools risk remaining academic exercises rather than becoming routine clinical aids.

Even so, AI will undoubtedly become an indispensable part of DFU research, promoting the development of microbiome-based personalized medicine and reducing the clinical burden of this refractory disease.

## 6 Conclusions and future perspectives

The refractory nature of DFUs cannot be explained by simple infection model; it is rooted in a vicious cycle between host pathology and microbial dysbiosis. This mini-review concludes that dysbiosis in both the gut and skin microbiomes drives ulcer chronicity. Systemically, disturbances to the gut microbiota via the gut-skin axis lead to a reduction in beneficial metabolites such as SCFAs and an accumulation of harmful ones like BCAAs. This results in persistent systemic inflammation and immune dysregulation, setting the stage for failed skin repair. Locally, the foot ulcer microenvironment suffers a significant loss of microbial diversity and becomes dominated by opportunistic pathogens such as *S. aureus* and *P. aeruginosa*, which form biofilms. These biofilms impede antibiotic penetration and induce metabolic dormancy in pathogens, enhancing tolerance. Concurrently, pathogen virulence factors directly damage tissue and exacerbate pre-existing immune cell dysfunction in diabetes, collectively disrupting the healing cascade. Therefore, the pathology of DFUs arises from a process of synergistic amplification, whereby host factors such as vascular disease, neuropathy, metabolic abnormalities, and immune dysregulation interact with and are reinforced by a dysbiotic microbiome.

Emerging therapeutic strategies aim to recalibrate the abnormal host-microbe interface by shifting the focus from broad-spectrum eradication to targeted ecological restoration. Promising avenues currently under investigation to modulate the gut-skin axis, disrupt biofilms, and restore a pro-healing microbiome include interventions such as probiotic supplementation, bacteriophage therapy, FMT, and TCM. These approaches utilize mechanisms such as competitive exclusion, virulence attenuation, and immunomodulation. However, the current evidence for most of these modalities is predominantly derived from preclinical studies, small-scale human trials, or case series. Consequently, their clinical integration requires robust validation through large-scale randomized controlled trials to definitively establish efficacy, optimal protocols, and long-term safety, a translational gap that remains substantial.

Future perspectives point towards a new era of personalized microbial medicine for the management of DFUs, jointly driven by multi-omics technologies and AI, pending systematic resolution of technical, validation, and implementation barriers. A key area of research is the development of AI-enabled multi-omics integration platforms to categorize patients based on their specific “microbial ecotype” and functional profile. This will enable targeted interventions, such as defined probiotic cocktails or precision phage therapy, to be matched to an individual’s dysbiotic signature. Meanwhile, research must focus on developing novel biofilm-disrupting agents and improving drug delivery to the wound bed, as well as translating AI analytical models and auxiliary diagnostic tools into routine clinical care. Combining antimicrobial strategies with modulators of the host’s immune and metabolic environment is a more holistic approach. If these challenges can be overcome, shifting the therapeutic goal from indiscriminate killing to ecological reconstruction and leveraging AI for precise intervention could significantly improve healing rates and reduce the global burden of DFU-related amputations and mortality.

## References

[B1] Argañaraz Aybar J. N. Ortiz Mayor S. Olea L. Garcia J. J. Nisoria S. Kolling Y. . (2022). Topical administration of Lactiplantibacillus plantarum accelerates the healing of chronic diabetic foot ulcers through modifications of infection, angiogenesis, macrophage phenotype and neutrophil response. Microorganisms 10 (3), 634. doi: 10.3390/microorganisms10030634 35336209 PMC8955315

[B2] Armstrong D. G. Tan T. W. Boulton A. J. M. Bus S. A. (2023). Diabetic foot ulcers: a review. Jama 330, 62–75. doi: 10.1111/iwj.70795 37395769 PMC10723802

[B3] Awasthi A. Corrie L. Vishwas S. Gulati M. Kumar B. Chellappan D. K. . (2022). Gut dysbiosis and diabetic foot ulcer: Role of probiotics. Pharmaceutics 14 (11), 2543. doi: 10.3390/pharmaceutics14112543 36432734 PMC9699533

[B4] Brocato J. Chervona Y. Costa M. (2014). Molecular responses to hypoxia-inducible factor 1α and beyond. Mol. Pharmacol. 85, 651–657. doi: 10.1124/mol.113.089623 24569087 PMC3990019

[B5] Bus S. A. Sacco I. C. N. Monteiro-Soares M. Raspovic A. Paton J. Rasmussen A. . (2024). Guidelines on the prevention of foot ulcers in persons with diabetes (IWGDF 2023 update). Diabetes Metab. Res. Rev. 40, e3651. doi: 10.1002/dmrr.3651 37302121

[B6] Cao Z. Wang F. Li X. Hu J. He Y. Zhang J. (2022). Characteristics of plantar pressure distribution in diabetes with or without diabetic peripheral neuropathy and peripheral arterial disease. J. Healthc Eng. 2022, 2437831. doi: 10.1155/2022/2437831 35707567 PMC9192305

[B7] Carrillo-Larco R. M. de Elvira Mori Orrillo E. Castillo-Cara M. García R. Yovera-Aldana M. Bernabe-Ortiz A. (2026). Identification of antibiotic resistance profiles in diabetic foot infections: A machine learning proof-of-concept analysis. Int. J. Diabetes Developing Countries 46, 193–200. doi: 10.1007/s13410-025-01490-1 30311153

[B8] Çelo E. Dama A. Hasho S. Deda L. (2026). Topical probiotics in diabetic wound healing: Emerging therapeutic strategies. Int. J. Mol. Sci. 27 (6), 2826. doi: 10.3390/ijms27062826 41898683 PMC13026683

[B9] Chen J. Qin S. Liu S. Zhong K. Jing Y. Wu X. . (2023). Targeting matrix metalloproteases in diabetic wound healing. Front. Immunol. 14, 2023. doi: 10.3389/fimmu.2023.1089001 36875064 PMC9981633

[B10] Dawi J. Tumanyan K. Tomas K. Misakyan Y. Gargaloyan A. Gonzalez E. . (2025). Diabetic foot ulcers: pathophysiology, immune dysregulation, and emerging therapeutic strategies. Biomedicines 13, 1076. doi: 10.3390/biomedicines13051076 40426903 PMC12109115

[B11] Deng L. Wang G. Ju S. (2024). Correlation between inflammatory factors, autophagy protein levels, and infection in granulation tissue of diabetic foot ulcer. Immun. Inflamm. Dis. 12, e1233. doi: 10.1002/iid3.1233 38577990 PMC10996373

[B12] Dörr S. Freier F. Schlecht M. Lobmann R. (2021). Bacterial diversity and inflammatory response at first-time visit in younger and older individuals with diabetic foot infection (DFI). Acta Diabetol. 58, 181–189. doi: 10.5005/jp/books/10922_10 32944830

[B13] Dörr S. Holland-Letz A. K. Weisser G. Chatzitomaris A. Lobmann R. (2023). Bacterial diversity, antibiotic resistance, and the risk of lower limb amputation in younger and older individuals with diabetic foot infection. Int. J. Low Extrem Wounds 22, 63–71. doi: 10.5005/jp/books/10922_10 33745353

[B14] Durand B. Yahiaoui Martinez A. Baud D. François P. Lavigne J. P. Dunyach-Remy C. (2022). Comparative genomics analysis of two Helcococcus kunzii strains co-isolated with Staphylococcus aureus from diabetic foot ulcers. Genomics 114, 110365. doi: 10.1016/j.ygeno.2022.110365 35413435

[B15] Esfandiari A. H. Mobarezi Z. Abolbashari S. Meshkat Z. (2025a). Efficacy of phage therapy in diabetic foot ulcers (DFUs): a systematic review. BMC Infect. Dis. 25, 819. doi: 10.1186/s12879-025-11258-x 40596887 PMC12211258

[B16] Esfandiari A. H. Mobarezi Z. Abolbashari S. Meshkat Z. (2025b). Efficacy of phage therapy in diabetic foot ulcers (DFUs): a systematic review. BMC Infect. Dis. 25, 819. doi: 10.1186/s12879-025-11258-x 40596887 PMC12211258

[B17] Feng J. Wang J. Wang Y. Huang X. Shao T. Deng X. . (2022). Oxidative stress and lipid peroxidation: prospective associations between ferroptosis and delayed wound healing in diabetic ulcers. Front. Cell Dev. Biol. 10, 2022. doi: 10.3389/fcell.2022.898657 35874833 PMC9304626

[B18] Gan Y. Zhang J. Qi F. Hu Z. Sweren E. Reddy S. K. . (2024). Commensal microbe regulation of skin cells in disease. Cell Host Microbe 32, 1264–1279. doi: 10.1016/j.chom.2024.07.020 39146798 PMC11457753

[B19] Garg S. S. Gupta J. (2022). Polyol pathway and redox balance in diabetes. Pharmacol. Res. 182, 106326. doi: 10.1016/j.phrs.2022.106326 35752357

[B20] Guan H. Wang Y. Niu P. Zhang Y. Zhang Y. Miao R. . (2024). The role of machine learning in advancing diabetic foot: A review. Front. Endocrinol. (Lausanne) 15, 1325434. doi: 10.3389/fendo.2024.1325434 38742201 PMC11089132

[B21] Hsiu-Jung L. Tzu-Chi L. Yu-Feng W. (2026). Integrating regenerative therapies and artificial intelligence for precision management of diabetic foot ulcers. Adv. Wound Care. 21621918261422388. doi: 10.1177/21621918261422388 41644477

[B22] Hu X. Zhou J. Guo M. Peng W. Yang C. Wang F. . (2026). Expression of lactylation-related genes and their correlation with diabetic foot ulcer occurrence and immune infiltration. Front. Immunol. 17, 1765123. doi: 10.3389/fimmu.2026.1765123 42136654 PMC13167497

[B23] Huang Z. Liu J. Zheng X. Geng X. Tan J. Ai Y. . (2026). Integrating bioinformatic prediction and the "gut microbiota-inflammation-skin axis" to decipher the mechanisms of quercetin (from Evodia rutaecarpa) in diabetic wound healing. Front. Immunol. 17, 1755280. doi: 10.3389/fimmu.2026.1755280 41816323 PMC12974265

[B24] Huang Y. Tang Y. Zhao X. Xu M. Chen M. (2025). Novel insights into the role of gut microbiota and its metabolites in diabetic chronic wounds. FASEB J. 39, e70316. doi: 10.1096/fj.202401478rr 39785136

[B25] Hussain A. Mojgani N. Shah S. M. A. Kousar N. Ali S. A. (2025). The emerging role of probiotics in the management and treatment of diabetic foot ulcer: A comprehensive review. AIMS Microbiol. 11, 649–678. doi: 10.3934/microbiol.2025027 41078547 PMC12511964

[B26] Jimenez-Sanchez M. Celiberto L. S. Yang H. Sham H. P. Vallance B. A. (2025). The gut-skin axis: a bi-directional, microbiota-driven relationship with therapeutic potential. Gut Microbes 17, 2473524. doi: 10.1080/19490976.2025.2473524 40050613 PMC11901370

[B27] Kalan L. R. Meisel J. S. Loesche M. A. Horwinski J. Soaita I. Chen X. . (2019). Strain- and species-level variation in the microbiome of diabetic wounds is associated with clinical outcomes and therapeutic efficacy. Cell Host Microbe 25, 641–655.e645. doi: 10.1016/j.chom.2019.03.006 31006638 PMC6526540

[B28] Karmińska M. Czernikiewicz K. Głowacka-Kamińska W. Skrzypska N. Wróblewska J. Wojtczak O. . (2026). Probiotic and postbiotic delivery systems for the management of chronic wounds: A review of emerging strategies. Cureus 18, e105081. doi: 10.7759/cureus.105081 41994838 PMC13081014

[B29] Liu S. Goonetilleke S. Hon K. Burdon I. A. Bouras G. S. McMillan N. . (2023). “ Bacteriophage in combination with ciprofloxacin against Pseudomonas aeruginosa infections in diabetic foot ulcer patients,” in Biorxiv (Cold Spring Harbor: Cold Spring Harbor Laboratory Press), 2023.2010. 2003.560795.

[B30] Liu Y. Yang Z. Zhou X. Li Z. Hideki N. (2024). Diacylglycerol kinases and its role in lipid metabolism and related diseases. Int. J. Mol. Sci. 25, 13207. doi: 10.3390/ijms252313207 39684917 PMC11643042

[B31] Lou J. Xiang Z. Zhu X. Li J. Jin G. Cui S. . (2025). Skin microbiota and diabetic foot ulcers. Front. Microbiol. 16, 1575081. doi: 10.3389/fmicb.2025.1575081 40666801 PMC12261678

[B32] Lu Z. An N. Sheng S. Hong M. Deng P. Wu J. . (2025). Machine learning combining external validation to explore the immunopathogenesis of diabetic foot ulcer and predict therapeutic drugs. PloS One 20, e0328906. doi: 10.1371/journal.pone.0328906 40749079 PMC12316216

[B33] Lv D. Cao X. Zhong L. Dong Y. Xu Z. Rong Y. . (2023). Targeting phenylpyruvate restrains excessive NLRP3 inflammasome activation and pathological inflammation in diabetic wound healing. Cell Rep. Med. 4, 101129. doi: 10.1016/j.xcrm.2023.101129 37480849 PMC10439185

[B34] Macdonald K. E. Boeckh S. Stacey H. J. Jones J. D. (2021). The microbiology of diabetic foot infections: a meta-analysis. BMC Infect. Dis. 21, 770. doi: 10.1186/s12879-021-06516-7 34372789 PMC8351150

[B35] MacGibeny M. A. Adjei S. Pyle H. Bunick C. G. Ghannoum M. Grada A. . (2025). The human skin microbiome in health. J. Am. Acad. Dermatol. 93, 329–336. doi: 10.1016/j.jaad.2024.07.1498 39168311 PMC11912297

[B36] McDermott K. Fang M. Boulton A. J. M. Selvin E. Hicks C. W. (2023). Etiology, epidemiology, and disparities in the burden of diabetic foot ulcers. Diabetes Care 46, 209–221. doi: 10.2337/dci22-0043 36548709 PMC9797649

[B37] Mihai M. M. Bălăceanu-Gurău B. Ion A. Holban A. M. Gurău C. D. Popescu M. N. . (2024). Host-microbiome crosstalk in chronic wound healing. Int. J. Mol. Sci. 25 (9), 4629. doi: 10.3390/ijms25094629 38731848 PMC11083077

[B38] Nie X. Jiang Y. Meng X. Liu J. Zhao H. Chen Y. . (2026). Development and external validation of a machine learning model for predicting wound infection in diabetic foot ulcers. Diabetes Metab. Syndr. Obes. 19, 586810. doi: 10.2147/dmso.s586810 41821605 PMC12978000

[B39] Omo-Okhuasuyi A. Jin Y. F. ElHefnawi M. Chen Y. Flores M. (2024). Multimodal identification of molecular factors linked to severe diabetic foot ulcers using artificial intelligence. Int. J. Mol. Sci. 25 (19), 10686. doi: 10.3390/ijms251910686 39409014 PMC11476782

[B40] Pan Y. Chen L. Chen Y. Thomas E. R. Zhou S. Yang Y. . (2025). Mitochondrial dysfunction in diabetic ulcers: pathophysiological mechanisms and targeted therapeutic strategies. Front. Cell Dev. Biol. 13, 1625474. doi: 10.3389/fcell.2025.1625474 40917755 PMC12408512

[B41] Park J. U. Oh B. Lee J. P. Choi M. H. Lee M. J. Kim B. S. (2019). Influence of microbiota on diabetic foot wound in comparison with adjacent normal skin based on the clinical features. BioMed. Res. Int. 2019, 7459236. doi: 10.1155/2019/7459236 31531366 PMC6720033

[B42] Patel B. K. Patel K. H. Huang R. Y. Lee C. N. Moochhala S. M. (2022). The gut-skin microbiota axis and its role in diabetic wound healing-a review based on current literature. Int. J. Mol. Sci. 23 (5), 2375. doi: 10.3390/ijms23042375 35216488 PMC8880500

[B43] Pavithra T. R. Kaliyaperumal R. (2026). Epidemiology, pathophysiology, and management of diabetic foot: a comprehensive review. Foot 66, 102225. doi: 10.1016/j.foot.2026.102225 41637846

[B44] Peng C. Lei P. Qi H. Zhu Q. Huang C. Fu J. . (2025). Effect of fecal microbiota transplantation on diabetic wound healing through the IL-17A-mTOR-HIF1α signaling axis. Appl. Environ. Microbiol. 91, e0201924. doi: 10.1128/aem.02019-24 40019272 PMC11921319

[B45] Pham H. T. Veves A. Xu R. Lyons T. (2025). Microcirculation of the diabetic foot. Clin. Podiatr Med. Surg. 42, 599–614. doi: 10.1016/j.cpm.2025.06.005 41101879

[B46] Plumet L. Magnan C. Costechareyre D. Sotto A. Lavigne J. P. Molle V. (2025). Phage therapy: a promising approach for Staphylococcus aureus diabetic foot infections. J. Virol. 99, e0045825. doi: 10.1128/jvi.00458-25 40366171 PMC12172479

[B47] Prajapati S. K. Lekkala L. Yadav D. Jain S. Yadav H. (2025). Microbiome and postbiotics in skin health. Biomedicines 13. doi: 10.3390/biomedicines13040791 40299368 PMC12025169

[B48] Qaed E. Aldahmash W. Mahyoub M. A. (2025). Advanced glycation end products (AGEs) and their role in diabetes mellitus and related complications: mechanisms and therapeutic insights. Glycoconjugate J. 42, 209–223. doi: 10.1007/s10719-025-10194-x 40982076

[B49] Sahoo K. Meshram S. (2024). Biofilm formation in chronic infections: a comprehensive review of pathogenesis, clinical implications, and novel therapeutic approaches. Cureus 16, e70629. doi: 10.7759/cureus.70629 39483571 PMC11527504

[B50] Sharma D. Misba L. Khan A. U. (2019). Antibiotics versus biofilm: an emerging battleground in microbial communities. Antimicrob. Resist. Infect. Control 8, 76. doi: 10.1186/s13756-019-0533-3 31131107 PMC6524306

[B51] Sharma H. Sharma S. Krishnan A. Yuan D. Vangaveti V. N. Malabu U. H. . (2022). The efficacy of inflammatory markers in diagnosing infected diabetic foot ulcers and diabetic foot osteomyelitis: systematic review and meta-analysis. PloS One 17, e0267412. doi: 10.1371/journal.pone.0267412 35476639 PMC9045669

[B52] Shrestha L. Fan H. M. Tao H. R. Huang J. D. (2022). Recent strategies to combat biofilms using antimicrobial agents and therapeutic approaches. Pathogens 11 (3), 292. doi: 10.3390/pathogens11030292 35335616 PMC8955104

[B53] Soldevila-Boixader L. Carrera-Salinas A. Mur I. Morata L. Rivera A. Bosch J. . (2025). Exploring the microbiome of diabetic foot ulcers: A focus on cases with a clinical worse outcome. Antibiotics 14, 724. doi: 10.3390/antibiotics14070724 40724025 PMC12291833

[B54] Srivastava P. Sondak T. Sivashanmugam K. Kim K. S. (2022). A review of immunomodulatory reprogramming by probiotics in combating chronic and acute diabetic foot ulcers (DFUs). Pharmaceutics 14 (11), 2436. doi: 10.3390/pharmaceutics14112436 36365254 PMC9699442

[B55] Suryaletha K. John J. Radhakrishnan M. P. George S. Thomas S. (2018). Metataxonomic approach to decipher the polymicrobial burden in diabetic foot ulcer and its biofilm mode of infection. Int. Wound J. 15, 473–481. doi: 10.1111/iwj.12888 29356343 PMC7949765

[B56] Tang M. Feng J. Ni Y. Zhang W. Zhou M. Zhao C. (2026). Branch-chain amino acid accumulation: multiple roles in the pathogenesis of diabetic foot ulcers. Diabetes Res. Clin. Pract. 236, 113255. doi: 10.1016/j.diabres.2026.113255 41962631

[B57] Tang X. Li G. Wang G. Chen S. Zhang L. (2026). Expert consensus on acupuncture for diabetic foot. Am. J. Clin. Exp. Immunol. 15, 28–36. doi: 10.62347/uqmi4547 41869496 PMC13000733

[B58] T N V. V. Premnath M. Stanley J. V. Paul N. Mathew J. Radhakrishnan E. K. (2025). Whole genome sequencing based prediction of antimicrobial resistance evolution among the predominant bacterial pathogens of diabetic foot ulcer. World J. Microbiol. Biotechnol. 41, 161. 40312599 10.1007/s11274-025-04362-2

[B59] Tsai W. H. Chou C. H. Huang T. Y. Wang H. L. Chien P. J. Chang W. W. . (2021). Heat-killed lactobacilli preparations promote healing in the experimental cutaneous wounds. Cells 10 (11), 3264. doi: 10.3390/cells10113264 34831486 PMC8625647

[B60] Vijay V. Senthil G. Bamila S. Secunda R. Raghul K. (2024). A clinical study to evaluate autofluorescence imaging of diabetic foot ulcers using a novel artificial intelligence enabled noninvasive device. Int. J. Lower Extremity Wounds 23, 169–176. doi: 10.1177/15347346211047098 34617810

[B61] Wang G. Lin Z. Li Y. Chen L. Reddy S. K. Hu Z. . (2023). Colonizing microbiota is associated with clinical outcomes in diabetic wound healing. Adv. Drug Delivery Rev. 194, 114727. doi: 10.1016/j.addr.2023.114727 36758858 PMC10163681

[B62] Wang Q. Liu C. An J. Liu J. Wang Y. Cai Y. (2025). Mechanisms of microbial infection and wound healing in diabetic foot ulcer: pathogenicity in the inflammatory-proliferative phase, chronicity, and treatment strategies. Front. Endocrinol. (Lausanne) 16, 1657928. doi: 10.3389/fendo.2025.1657928 41103650 PMC12520920

[B63] Wang J. Wang K. Hu Z. Wang Y. Kang Y. Liu X. . (2026). Role of lactylation-induced macrophage failed phenotypic switching in sustaining inflammation of diabetic wounds. Front. Immunol. 17, 2026. doi: 10.3389/fimmu.2026.1777272 41869297 PMC13002385

[B64] Wang Y. Zhang H. Ma G. Tian Z. Wang B. (2022). The contribution of intestinal Streptococcus to the pathogenesis of diabetic foot ulcers: an analysis based on 16S rRNA sequencing. Int. Wound J. 19, 1658–1668. 35112796 10.1111/iwj.13766 PMC9615275

[B65] Woldeteklie A. A. Kebede H. B. Abdela A. A. Woldeamanuel Y. (2022). Prevalence of extended-spectrum β-lactamase and carbapenemase producers of gram-negative bacteria, and methicillin-resistant Staphylococcus aureus in isolates from diabetic foot ulcer patients in Ethiopia. Infect. Drug Resist. 15, 4435–4441. doi: 10.2147/idr.s371431 35978723 PMC9377397

[B66] Wolf S. J. Melvin W. J. Gallagher K. (2021). Macrophage-mediated inflammation in diabetic wound repair. Semin. Cell Dev. Biol. 119, 111–118. doi: 10.1016/j.semcdb.2021.06.013 34183242 PMC8985699

[B67] Young M. J. Hall L. M. L. Merabishvilli M. Pirnay J.-P. Clark J. R. Jones J. D. (2023). Phage therapy for diabetic foot infection: A case series. Clin. Ther. 45, 797–801. doi: 10.1016/j.clinthera.2023.06.009 37442654

[B68] Zafer M. M. Mohamed G. A. Ibrahim S. R. M. Ghosh S. Bornman C. Elfaky M. A. (2024). Biofilm-mediated infections by multidrug-resistant microbes: a comprehensive exploration and forward perspectives. Arch. Microbiol. 206, 101. doi: 10.1007/s00203-023-03826-z 38353831 PMC10867068

[B69] Zakir M. Ahuja N. Surksha M. A. Sachdev R. Kalariya Y. Nasir M. . (2023). Cardiovascular complications of diabetes: from microvascular to macrovascular pathways. Cureus 15, e45835. doi: 10.7759/cureus.45835 37881393 PMC10594042

[B70] Zhang Y. Sun X. Wu M. Tang X. (2025). Boruta algorithm-guided antibiotic selection in antibiotic-loaded bone cement for diabetic foot ulcers: microbiota and susceptibility analysis. Front. Pharmacol. 16, 1677198. doi: 10.3389/fphar.2025.1677198 41064447 PMC12500687

[B71] Zhou S. Hu X. Wang Y. Fei W. Sheng Y. Que H. (2024). The global prevalence of methicillin-resistant Staphylococcus aureus in patients with diabetic foot ulcers: a systematic review and meta-analysis. Diabetes Metab. Syndr. Obes. 17, 563–574. doi: 10.2147/dmso.s446911 38333763 PMC10849909

[B72] Zhou N. Shi L. Yuan X. Lang J. Wang J. Li J. . (2025). Uncovering the gut - skin axis: The role of specific traditional Chinese medicine interventions in regulating gut microbiota for diabetic foot ulcers and the analysis of research status. Front. Pharmacol. 16, 1641036. doi: 10.3389/fphar.2025.1641036 41079734 PMC12507883

